# Effects of Hypoxia in Intestinal Tumors on Immune Cell Behavior in the Tumor Microenvironment

**DOI:** 10.3389/fimmu.2021.645320

**Published:** 2021-03-02

**Authors:** Luping Zhang, Shaokun Wang, Yachen Wang, Weidan Zhao, Yingli Zhang, Nan Zhang, Hong Xu

**Affiliations:** ^1^Department of Gastroenterology, The First Hospital of Jilin University, Changchun, China; ^2^Department of Emergency, The First Hospital of Jilin University, Changchun, China

**Keywords:** colorectal cancer, hypoxia, immune cell infiltration, tumor microenvironment, risk score

## Abstract

**Background:**

Imbalanced nutritional supply and demand in the tumor microenvironment often leads to hypoxia. The subtle interaction between hypoxia and immune cell behavior plays an important role in tumor occurrence and development. However, the functional relationship between hypoxia and the tumor microenvironment remains unclear. Therefore, we aimed to investigate the effect of hypoxia on the intestinal tumor microenvironment.

**Method:**

We extracted the names of hypoxia-related genes from the Gene Set Enrichment Analysis (GSEA) database and screened them for those associated with colorectal cancer prognosis, with the final list including *ALDOB*, *GPC1*, *ALDOC*, and *SLC2A3*. Using the sum of the expression levels of these four genes, provided by The Cancer Genome Atlas (TCGA) and Gene Expression Omnibus (GEO) databases, and the expression coefficients, we developed a hypoxia risk score model. Using the median risk score value, we divided the patients in the two databases into high- and low-risk groups. GSEA was used to compare the enrichment differences between the two groups. We used the CIBERSORT computational method to analyze immune cell infiltration. Finally, the correlation between these five genes and hypoxia was analyzed.

**Result:**

The prognosis of the two groups differed significantly, with a higher survival rate in the low-risk group than in the high-risk group. We found that the different risk groups were enriched by immune-related and inflammatory pathways. We identified activated M0 macrophages in TCGA and GEO databases and found that *CCL2/4/5*, and *CSF1* contributed toward the increased infiltration rate of this immune cell type. Finally, we observed a positive correlation between the five candidate genes’ expression and the risk of hypoxia, with significant differences in the level of expression of each of these genes between patient risk groups.

**Conclusion:**

Overall, our data suggest that hypoxia is associated with the prognosis and rate of immune cell infiltration in patients with colorectal cancer. This finding may improve immunotherapy for colorectal cancer.

## Introduction

Since Stephen Paget proposed the “seed and soil” theory of cancer development and tumor metastasis (1), the understanding of the tumor microenvironment has gradually deepened. Several components of the tumor microenvironment contribute toward tumor occurrence and development (2, 3). An imbalance between nutrient supply and demand within the tumor often leads to hypoxia, glucose deficiency, and consequently an acidic tumor microenvironment (4). Specifically, tumor cells can use immune escape mechanisms to drive metastasis and invasion in anoxic environments (5). This has increased research interest into the relationship between hypoxia and the tumor immune microenvironment.

Hypoxia can reduce the activity of various immune cells in the tumor microenvironment and the production of corresponding immune stimulators to increase the release of suppressors and the expression of immune checkpoint inhibitors (5, 6), suggesting a close relationship between hypoxia and immune cells.

This study aimed to investigate the relationship between hypoxia-related genes and the immune microenvironment of colorectal cancer by (1) quantifying the influence of these genes on the tumor immune microenvironment, (2) screening hypoxia-related genes associated with intestinal tumor prognosis, (3) developing a hypoxia risk score model, (4) validating the model’s ability to predict patient prognosis and risk using data from The Cancer Genome Atlas (TCGA) and Gene Expression Omnibus (GEO) databases, (5) identifying the enrichment of model-related pathways, (6) using the hypoxia risk score as the entry point to explore differences in the infiltration rate of immune cells, and (7) identifying genes that affect immune cell infiltration to evaluate the relationship between hypoxia risk score and the tumor immune microenvironment.

## Materials and methods

### Raw Data

The intestinal cancer-related RNA-sequencing and clinical data used in this study were obtained from TCGA (https://portal.gdc.cancer.gov/) and GEO (gse39582) databases.

### Construction and Grouping of the Hypoxia Model

The screening of hypoxia-related genes identified the genes independently related to the prognosis of intestinal cancer. We then multiplied the expression levels of each gene in TCGA and GEO databases by their respective expression coefficients, with the resulting sum defined as the risk score for each patient. Based on the median risk score value, the patients in the two databases were divided into high- and low-risk groups for the follow-up evaluations.

### Survival Analysis

The survival and survminer R packages (The R Foundation for Statistical Computing, Vienna, Austria) were used to analyze the prognosis of 445 and 579 patients in TCGA and GEO databases, respectively. Patients in TCGA database were followed up for 12 years, while those in the GEO database were followed up for 16 years. The Kaplan–Meier method was used to plot the survival curves, and the log-rank test was used to evaluate the statistical significance between them, with p-values <0.05 considered statistically significant.

### ROC Curve Analysis

**Figure 2:**
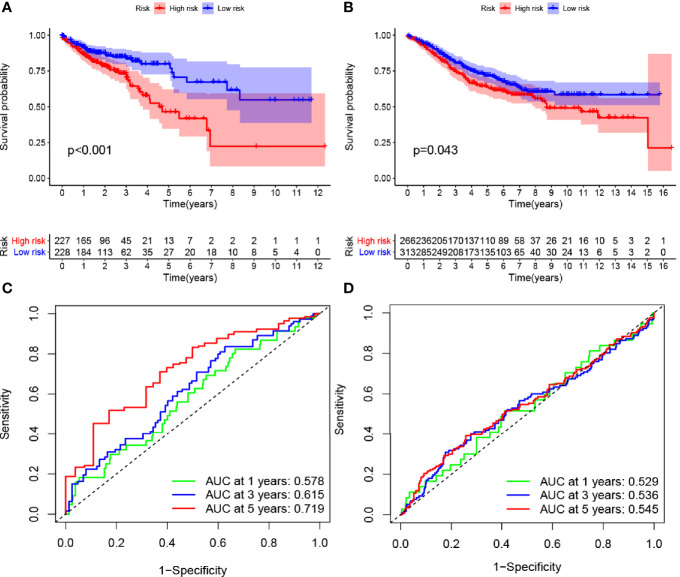
Effects of the hypoxia model on patient prognosis. **(A, B)** Kaplan–Meier survival curves for patients with colon cancer in The Cancer Genome Atlas and Gene Expression Omnibus databases, stratified according to risk scores (high vs. low); comparisons of the median survival time in both groups with log-rank tests (p<0.01 and p = 0.043, respectively). **(C, D)** Receiver operating characteristic curve analysis of the prognostic accuracy of the model.

ROC curve analysis was conducted using the survival, survminer, and timeROC packages in R. The 1-, 3-, and 5-year survival rates of patients in TCGA and GEO databases were evaluated. The area under the ROC curve for the 1-, 3-, and 5-year survival rates increased gradually and exceeded 0.5, which was defined as the threshold for the accurate prediction of survival by the model. Survival for each group is shown as risk columns and risk curves in **Figures 2A, B**.

### Heat Maps

In this study, heat maps of gene expression were drawn using the pheatmap package in R.

### PPI Network Analysis

A network of anoxic genes was constructed using the STRING database. R software was used to select the 50 genes with the largest numbers of adjacent nodes for subsequent analysis.

### Cox Regression Analysis

We applied the “survival” package in R and performed univariate Cox regression analysis to identify hypoxia-related genes that were closely related to prognosis. Univariate and multivariate prognostic analyses included factors such as age, sex, TNM stage, and the proposed risk score.

### Correlations Between Gene Expression and Hypoxia Risk

After screening for genes that play a key role in immune cell regulation, the ggplot2, GGPUBR, and ggExtra packages in R were used to analyze the correlations between gene expression and the risk of hypoxia, and the differences in expression levels between patients at high- and low-risk of hypoxia.

### Gene Set Enrichment Analysis

We downloaded the HALLMARK gene set and gene symbols from the GSEA website (https://www.gsea-msigdb.org/gsea/index.jsp) to extract hypoxia-related genes. The entire transcriptome of all tumor samples was used for GSEA, and only gene sets with nominal p-values <0.05 and FDR q values <0.06 were considered significant.

## Results

### Extraction and Screening of Hypoxia-Related Genes

**Figure 1:**
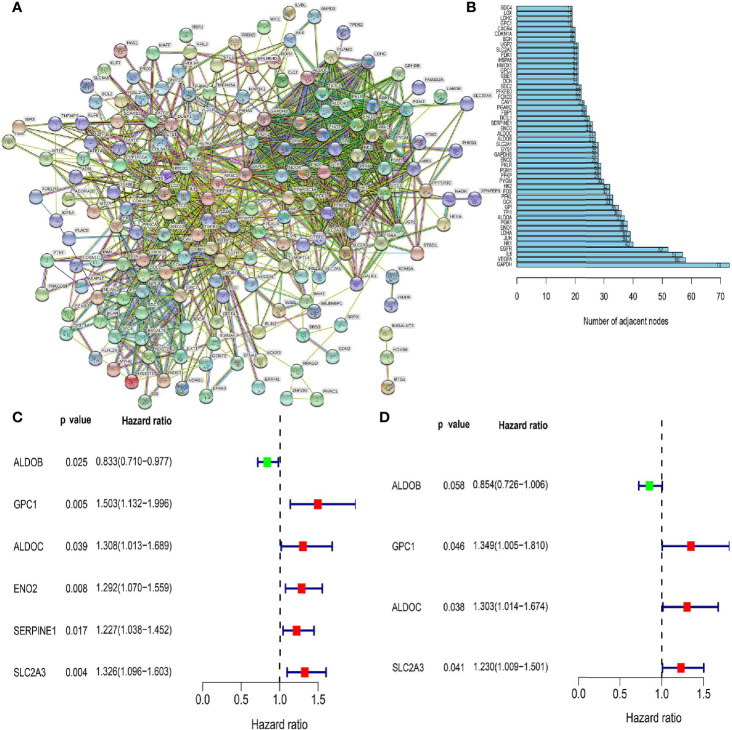
Screening for hypoxia-related genes and their relationship with patient prognosis. **(A)** Protein-protein interaction network containing members with interaction confidence values >0.4. **(B)** The top 50 genes selected based on the number of nodes and their sub-nodes. **(C)** Univariate Cox regression analysis identified candidate genes with p-values <0.05. **(D)** Among the genes related to colorectal cancer prognosis, the genes shown are those independently related to patient prognosis in a multifactor prognostic model of hypoxia.

We first downloaded the HALLMARK gene sets. The gene symbol set was obtained from the Gene Set Enrichment Analysis (GSEA) website (https://www.gsea-msigdb.org/gsea/index.jsp), which provides the names of all hypoxia-related genes. We then used the Search Tool for the Retrieval of Interacting Genes/Proteins (STRING) (http://string-db.org/cgi/input.pl), a protein-protein interaction (PPI) network database, to construct a model of PPIs between hypoxia-related genes (**Figure 1A**). By counting the number of adjacent nodes for each protein, we identified the core genes. The top 50 core genes with adjacent nodes are presented in **Figure 1B**. We then downloaded the intestinal tumor gene expression data and associated clinical information available from TCGA database. We extracted the data on hypoxia-related gene expression and screened genes associated with prognosis using univariate Cox regression analysis (**Figure 1C**). Among the identified genes, *ALDOB* was associated with a low risk, while five genes (*GPC1, ALDOC, ENO2, SERPINE1*, and *SLC2A3*) were associated with a high risk of developing tumor malignancy. The multivariate Cox regression analysis of genes associated with patient prognosis revealed four genes (**Figure 1D**) that were then used to construct prognostic models. These four genes (*ALDOB*, *GPC1, ALDOC*, and *SLC2A3*) had model coefficients of −0.1574, 0.2994, 0.2647, and 0.2074, respectively.

### Effects of Hypoxia-Related Genes on Prognosis

**Figure 3:**
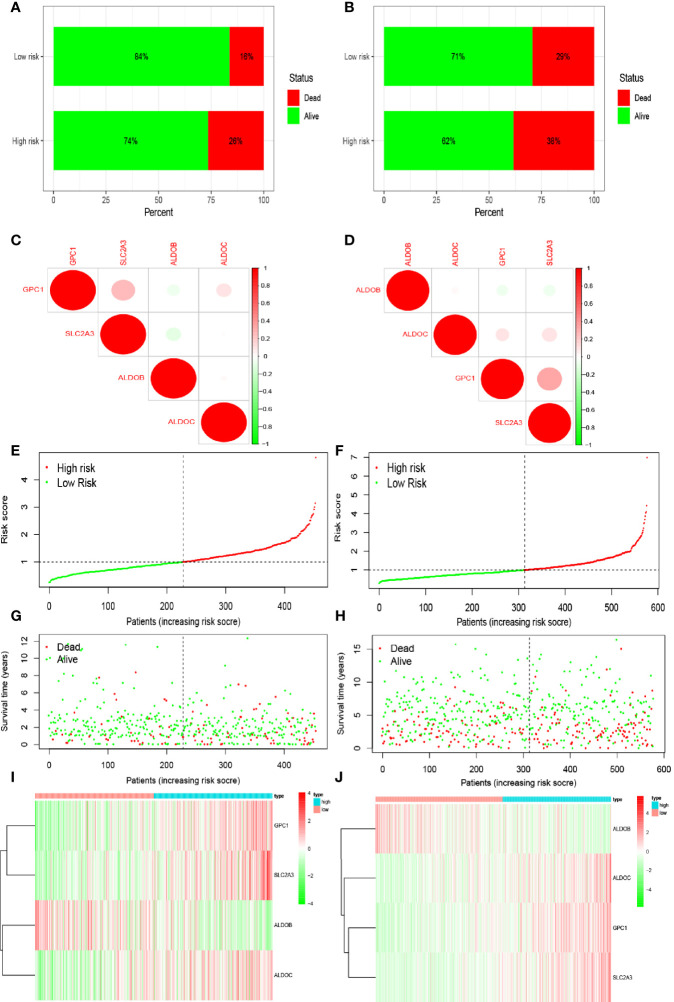
Prediction of patient risk in the hypoxia model and the expression levels of the genes included in the model. **(A, B)** Patient survival in The Cancer Genome Atlas (TCGA) and Gene Expression Omnibus (GEO) databases. **(C, D)** Correlations between the genes included in the risk model based on TCGA and GEO databases. Positive and negative correlations are indicated in red and green, respectively. **(E, F)** Patient risk scores in TCGA and GEO databases. **(G, H)** Survival rates in the high- and low-risk patient groups in TCGA and GEO databases. **(I, J)** Heat maps of gene expression levels in the risk model for the high- and low-risk groups in TCGA and GEO databases.

Multivariate Cox regression analysis identified four hypoxia-related genes associated with intestinal tumor prognosis, which were used for modeling. We multiplied the expression levels of these genes (as reported in TCGA and GEO databases) by the corresponding coefficients to obtain the risk score for each patient. The median risk score value was then used to divide the patients in TCGA database into high- and low-risk groups. The subsequent survival analysis revealed significant differences between the high- and low-risk groups (p < 0.05; **Figures 2A, B**). Receiver operating characteristic (ROC) curve analysis was used to verify the accuracy of the survival estimates derived from the present model, showing a gradually increasing accuracy of predicting the 1-, 3-, and 5-year survival rates of patients included in TCGA database (**Figure 2C**). The area under the ROC curve for the GEO database was > 0.05 (**Figure 2D**), indicating that the model accurately predicted the survival rate. To value the survival rate of patients more intuitively in the high- and low-risk groups, we used risk histograms to show the differences in survival status between the two databases (**Figures 3A, B**). We observed a higher proportion of surviving patients in the low-risk group than in the high-risk group. These results further demonstrated that our model effectively distinguished between high- and low-risk patients. We also analyzed the interactions between the hypoxia-related genes that were identified as affecting patient prognosis in the model (**Figures 3C, D**). We further demonstrated the relationship between patient risk and survival using risk curves, in which the risk scores for both groups of patients were plotted (**Figures 3E, F**). We observed a longer survival time in the low-risk group than in the high-risk group. Moreover, the number of deaths in the low-risk group decreased over time (**Figures 3G, H**). Finally, we compared the expression level of each gene included in the model between the high- and low-risk groups using thermography (**Figures 3I, J**).

### Effects of Different Clinical Characteristics on Intestinal Tumor Prognosis

**Figure 4:**
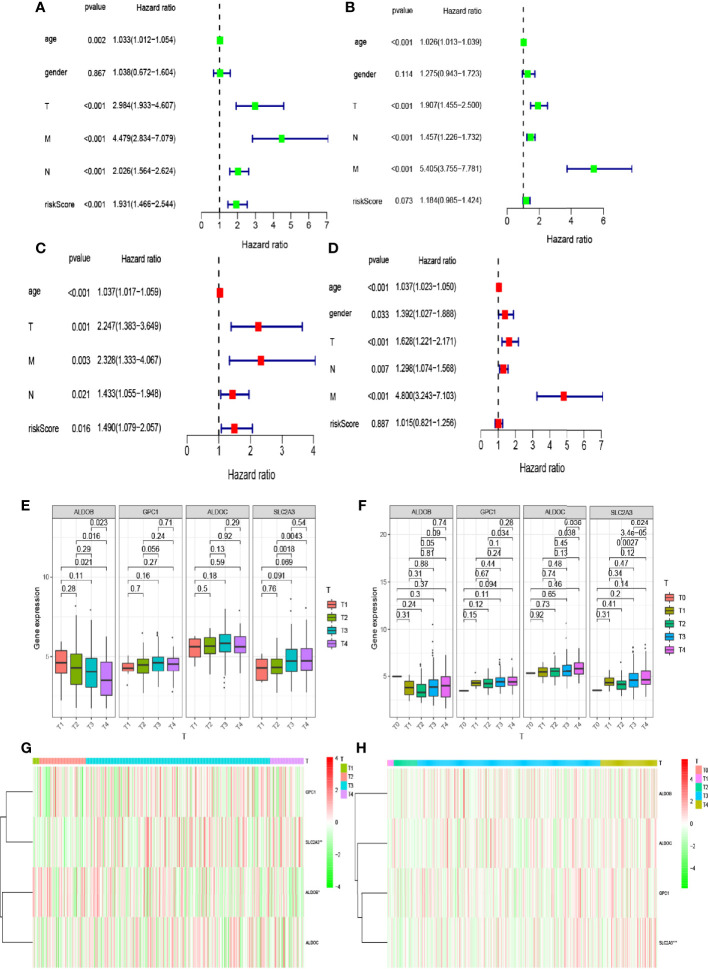
Relationship between the risk model and clinical factors. **(A, B)** Single-factor prognostic analysis included age, sex, tumor–node–metastasis (TNM) stage, and the risk scores of patients with colorectal cancer in The Cancer Genome Atlas (TCGA) and Gene Expression Omnibus (GEO) databases. **(C, D)** Multifactor prognostic analysis included age, sex, TNM stage, and the risk scores of patients with colorectal cancer in TCGA and GEO databases. **(E, F)** Comparisons of the expression levels of various genes in the hypoxia model in TCGA and GEO databases for different T stages. **(G, H)** Heat maps showing the expression levels of genes in the risk model in TCGA and GEO databases for different T stages.

Clinical characteristics differ in the impact they have on patient prognosis. Thus, we analyzed the impact of clinical characteristics on the prognosis of patients included in TCGA and GEO databases. We first used univariate Cox regression analysis to evaluate the impact of clinical characteristics on the survival time and prognosis of patients included in the two databases (**Figures 4A, B**). We found that patient sex affected neither survival nor prognosis, while other factors affected survival and prognosis and were associated with increased risk. However, the p-value of the risk score in our model was <0.05 for patients in TCGA database, indicating that the risk score also affected patient prognosis and survival. In contrast, the p-value was >0.05 for patients in the GEO database. Multivariate analysis of these factors showed that the p-values for age and tumor–node–metastasis (TNM) stage were both <0.05, indicating that these variables were independent prognostic factors (**Figures 4C, D**). We also observed differences in the expression levels of hypoxia-related genes between patients with different T stages included in the two databases (**Figures 4E–H**). The expression level of *SLC2A3* in different T stages (T1, T2, T3, and T4) differed significantly between patients in TCGA and GEO databases (p < 0.05).

### Enrichment of Pathways in Hypoxia-Related Risk Groups

**Figure 5:**
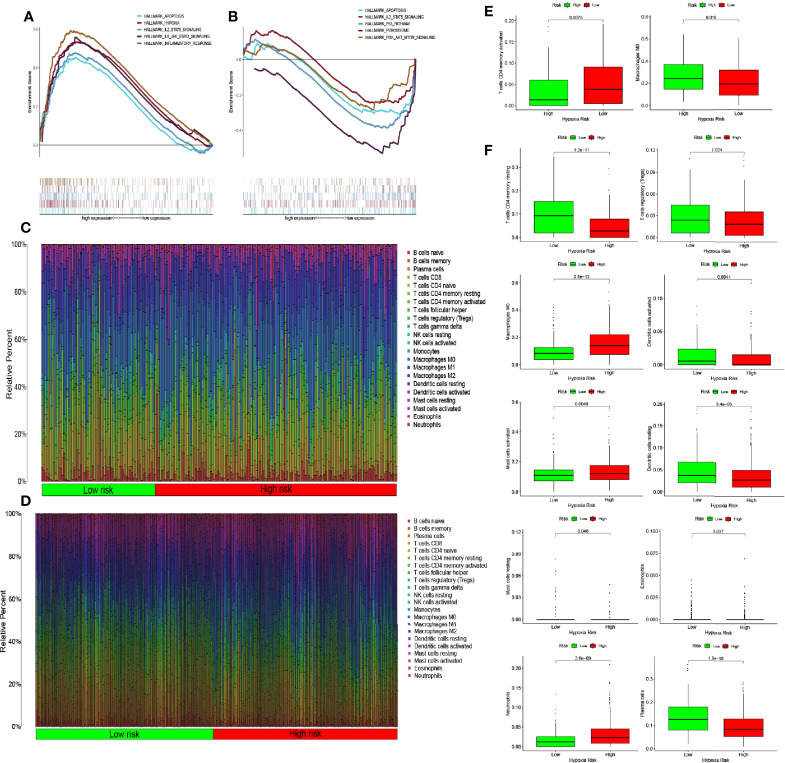
Enrichment of hypoxia pathways and infiltration of hypoxia-related immune cells. **(A)** Enriched gene sets in the HALLMARK collection according to high-risk scores in The Cancer Genome Atlas (TCGA) database. Each line represents one particular gene set with a unique color, with upregulated genes appearing on the left side approaching the origin of the coordinates and downregulated genes appearing on the right side of the x-axis. Only gene sets with nominal (NOM) p-values <0.05 and false discovery rate (FDR) q values <0.06 were considered statistically significant. A selection of leading gene sets is shown in the plot. **(B)** The enriched gene sets in the HALLMARK collection by low-risk scores in the Gene Expression Omnibus (GEO) database. Only gene sets with NOM p-values <0.05 and FDR q values of <0.06 were considered statistically significant. A selection of leading gene sets is shown in the plot. **(C, D)** Heat map of hypoxia risk and immune cell infiltration in TCGA and GEO databases. **(E)** Immune cells whose infiltration is significantly associated with the risk of hypoxia in TCGA database (p < 0.05). **(F)** Immune cells whose infiltration is significantly associated with the risk of hypoxia in the GEO database (p < 0.05).

We observed differences in the enrichment level of hypoxia-related genes and associated pathways between the high- and low-risk groups. To understand the level of pathway enrichment, we used GSEA software (UC San Diego, San Diego, CA, USA; Broad Institute, Cambridge, MA, USA) to compare the pathways between different risk groups. The high-risk group in TCGA database showed many enriched pathways related to apoptotic and immune functions (**Figure 5A**) compared with the low-risk group. Among these were apoptosis, hypoxia, interleukin (IL) 2/signal transducer and activator of transcription (STAT) 5 signaling, IL6-STAT3 signaling, and the inflammatory response pathways. The other related pathways are shown in **Table 1**. In contrast, the pathways enriched in the low-risk group mainly included those associated with oxidative phosphorylation and lipid metabolism, among others. The pathways with false discovery rate (FDR) q values >0.05 are shown in **Table 2**. The low-risk group of patients in the GEO database also showed the enrichment of apoptosis and IL2-STAT5 signaling pathways, in addition to, the p53 and peroxisome and phosphatidylinositol 3-kinase-mammalian target of rapamycin serine/threonine protein kinase B signaling pathways (**Figure 5B**). The enrichment of other pathways is shown in **Table 3**. The high-risk group also showed the enrichment of the Hedgehog and Wnt/beta-catenin signaling pathways. Pathways with FDR values >0.05 are shown in **Table 4**.

**Table 1:** Pathway enrichment in the group of patients at high risk of hypoxia in The Cancer Genome Atlas database.

NAME	SIZE	ES	NES	NOM p-val	FDR q-val	FWER p-val
MYOGENESIS	199	0.64095765	2.2988102	0	0	0
EPITHELIAL_MESENCHYMAL_TRANSITION	198	0.7808052	2.2663856	0	9.73E-04	0.001
COAGULATION	138	0.5881612	2.2491221	0	6.49E-04	0.001
APICAL_JUNCTION	200	0.5725273	2.210138	0	7.86E-04	0.002
HYPOXIA	197	0.5341002	2.1750667	0	0.001029189	0.003
ANGIOGENESIS	36	0.7042173	2.1259532	0	0.002047737	0.007
KRAS_SIGNALING_UP	199	0.53099084	2.0836837	0	0.003663993	0.015
UV_RESPONSE_DN	142	0.5923043	2.0315046	0.001988072	0.00553734	0.022
HEDGEHOG_SIGNALING	36	0.6280092	2.0067165	0.003913894	0.005807516	0.026
RESPONSE	200	0.5912703	2.0001185	0.003738318	0.005448199	0.027
COMPLEMENT	200	0.5222255	1.9398772	0.001926782	0.008785588	0.045
IL2_STAT5_SIGNALING	199	0.47638285	1.9347376	0.001923077	0.008227405	0.047
NOTCH_SIGNALING	32	0.53026545	1.8844135	0.005928854	0.011401303	0.078
APICAL_SURFACE	44	0.52808076	1.8692672	0	0.012468602	0.085
APOPTOSIS	159	0.45459515	1.8656777	0.003846154	0.012308807	0.089
TNFA_SIGNALING_VIA_NFKB	199	0.5475131	1.8406801	0.009380863	0.014613844	0.114
IL6_JAK_STAT3_SIGNALING	87	0.5565036	1.7961316	0.0056926	0.020605113	0.158
TGF_BETA_SIGNALING	54	0.47515002	1.6418401	0.034951456	0.059671924	0.381
ALLOGRAFT_REJECTION	196	0.5172241	1.5929213	0.06	0.07578372	0.456
WNT_BETA_CATENIN_SIGNALING	42	0.47698218	1.5757742	0.045454547	0.079787806	0.484
ESTROGEN_RESPONSE_EARLY	198	0.32408136	1.4306225	0.055984557	0.15474646	0.701
CHOLESTEROL_HOMEOSTASIS	73	0.38557705	1.4078543	0.10685484	0.16225821	0.734
KRAS_SIGNALING_DN	199	0.34252158	1.3754886	0.07129095	0.17926803	0.779
UV_RESPONSE_UP	156	0.28952506	1.2597458	0.12741312	0.27369708	0.891
INTERFERON_GAMMA_RESPONSE	198	0.4217492	1.2542942	0.2848723	0.2676427	0.894
MITOTIC_SPINDLE	198	0.31854162	1.188202	0.31697342	0.3293753	0.935
P53_PATHWAY	195	0.27196786	1.1832048	0.21314742	0.3224601	0.937
ADIPOGENESIS	198	0.27874216	1.1698754	0.27734375	0.32397935	0.943
GLYCOLYSIS	199	0.26324964	1.1427033	0.26070037	0.34104726	0.956
ANDROGEN_RESPONSE	99	0.30673286	1.1124011	0.33870968	0.36284208	0.966
ESTROGEN_RESPONSE_LATE	198	0.2458808	1.1081452	0.2615694	0.35552585	0.967
HEME_METABOLISM	193	0.23562211	1.0899739	0.30113637	0.36277255	0.972
MTORC1_SIGNALING	197	0.24964637	0.90750676	0.5214724	0.5825153	0.997
PI3K_AKT_MTOR_SIGNALING	105	0.209095	0.88849974	0.634	0.59227496	0.997
PANCREAS_BETA_CELLS	40	0.23578233	0.70691955	0.8606061	0.83144087	1
INTERFERON_ALPHA_RESPONSE	96	0.19630556	0.54184294	0.8392157	0.95013	1

**Table 2:** Pathway enrichment in the group of patients at low risk of hypoxia in The Cancer Genome Atlas database.

NAME	SIZE	ES	NES	NOM p-val	FDR q-val	FWER p-val
OXIDATIVE_PHOSPHORYLATION	200	-0.6354224	-1.8517317	0.02	0.09670171	0.108
MYC_TARGETS_V1	196	-0.55293024	-1.5802475	0.091976516	0.29567894	0.452
E2F_TARGETS	198	-0.5320485	-1.5347565	0.115830116	0.25026736	0.533
FATTY_ACID_METABOLISM	156	-0.37549332	-1.483107	0.078125	0.24727894	0.605
PEROXISOME	104	-0.34722096	-1.4177548	0.08267716	0.26794004	0.708
BILE_ACID_METABOLISM	112	-0.33064038	-1.4166067	0.052529182	0.22491534	0.711
XENOBIOTIC_METABOLISM	197	-0.23699556	-1.0814732	0.31225297	0.6670434	0.973
MYC_TARGETS_V2	58	-0.4065364	-1.0632117	0.4333996	0.6178284	0.977
G2M_CHECKPOINT	195	-0.33763614	-1.0606128	0.425	0.55348146	0.978
DNA_REPAIR	150	-0.28392524	-1.0543846	0.39078155	0.50790316	0.981
PROTEIN_SECRETION	96	-0.30276886	-1.0146313	0.45114344	0.5174334	0.986
REACTIVE_OXYGEN_SPECIES_PATHWAY	49	-0.27658382	-0.960691	0.49802372	0.5441408	0.99
SPERMATOGENESIS	133	-0.2188226	-0.8435639	0.7213439	0.67120385	0.999
UNFOLDED_PROTEIN_RESPONSE	110	-0.18729709	-0.7100693	0.8192771	0.8042398	1

**Table 3:** Pathway enrichment in the group of patients at high risk of hypoxia in the Gene Expression Omnibus database.

NAME	SIZE	ES	NES	NOM p-val	FDR q-val	FWER p-val
HEDGEHOG_SIGNALING	35	0.47144	1.277345	0.14038461	1	0.749
WNT_BETA_CATENIN_SIGNALING	41	0.430082	1.2600657	0.15187377	0.96750426	0.763
EPITHELIAL_MESENCHYMAL_TRANSITION	195	0.5649699	1.176124	0.33539096	0.9005055	0.86
ANGIOGENESIS	35	0.46240142	1.0144869	0.45738044	1	0.956
MYOGENESIS	196	0.3335677	0.9412718	0.5278351	1	0.983
UV_RESPONSE_DN	135	0.3387805	0.9249301	0.5495868	0.99989307	0.985
TGF_BETA_SIGNALING	52	0.33623004	0.9151217	0.5373737	0.88053614	0.985
KRAS_SIGNALING_DN	190	0.263007	0.8919948	0.642562	0.81584287	0.987
APICAL_SURFACE	43	0.27583465	0.7981121	0.813278	0.91715556	0.996
KRAS_SIGNALING_UP	192	0.30013293	0.7668628	0.806	0.8810992	0.997
ANDROGEN_RESPONSE	93	0.22268894	0.7156268	0.86470586	0.8806738	0.998
COAGULATION	136	0.2703017	0.67810416	0.9207317	0.8534861	0.998

**Table 4:** Pathway enrichment in the group of patients at low risk of hypoxia in the Gene Expression Omnibus database.

NAME	SIZE	ES	NES	NOM p-val	FDR q-val	FWER p-val
OXIDATIVE_PHOSPHORYLATION	180	-0.5181038	-1.8664485	0.017612524	0.052985493	0.035
FATTY_ACID_METABOLISM	153	-0.5680401	-1.8461372	0.00203666	0.03559099	0.046
PEROXISOME	101	-0.53683174	-1.8330911	0	0.029562779	0.051
CHOLESTEROL_HOMEOSTASIS	71	-0.54806143	-1.6309602	0.021276595	0.15649396	0.239
ADIPOGENESIS	189	-0.41682148	-1.5094645	0.035433073	0.32561186	0.449
XENOBIOTIC_METABOLISM	193	-0.52328324	-1.5083747	0.028985508	0.2722546	0.449
REACTIVE_OXYGEN_SPECIES_PATHWAY	47	-0.4019267	-1.376308	0.11977186	0.51835406	0.644
GLYCOLYSIS	194	-0.39022183	-1.3331028	0.12045889	0.56736577	0.716
P53_PATHWAY	188	-0.3891394	-1.2930824	0.124756336	0.61373806	0.757
BILE_ACID_METABOLISM	109	-0.45229995	-1.2606792	0.17364016	0.63521326	0.791
ESTROGEN_RESPONSE_LATE	197	-0.3940817	-1.2011873	0.156	0.73189086	0.858
UV_RESPONSE_UP	153	-0.34357396	-1.175342	0.19662921	0.73222524	0.88
MYC_TARGETS_V2	56	-0.4696598	-1.1727189	0.37304688	0.6814103	0.88
MTORC1_SIGNALING	189	-0.38030684	-1.1696959	0.29032257	0.6386857	0.882
PI3K_AKT_MTOR_SIGNALING	103	-0.29268706	-1.132223	0.27868852	0.6835487	0.904
ESTROGEN_RESPONSE_EARLY	193	-0.32482004	-1.0662084	0.33464566	0.79412293	0.936
PANCREAS_BETA_CELLS	40	-0.42545584	-1.057426	0.38690478	0.767329	0.94
HEME_METABOLISM	189	-0.2667636	-1.0508605	0.36575875	0.7411266	0.942
DNA_REPAIR	144	-0.298164	-1.0362988	0.42330098	0.73253185	0.943
APOPTOSIS	159	-0.3165646	-0.9227984	0.57114625	0.955858	0.981
SPERMATOGENESIS	132	-0.24104334	-0.86871046	0.6871287	1	0.988
COMPLEMENT	194	-0.32908297	-0.8492691	0.6442308	1	0.991
APICAL_JUNCTION	188	-0.27416155	-0.83836824	0.64705884	1	0.992
MYC_TARGETS_V1	185	-0.271399	-0.80545324	0.628	1	0.995
INTERFERON_GAMMA_RESPONSE	194	-0.3529506	-0.7729238	0.6927593	1	0.996
INTERFERON_ALPHA_RESPONSE	92	-0.36440378	-0.7677859	0.6827853	1	0.996
ALLOGRAFT_REJECTION	193	-0.32352465	-0.7621619	0.69921875	1	0.996
IL2_STAT5_SIGNALING	193	-0.24943894	-0.74676377	0.8172888	1	0.997
UNFOLDED_PROTEIN_RESPONSE	103	-0.20758167	-0.70057595	0.7745665	1	0.998
TNFA_SIGNALING_VIA_NFKB	194	-0.30261576	-0.6995795	0.80670613	1	0.998
HYPOXIA	190	-0.24055389	-0.6963746	0.8644401	1	0.998
MITOTIC_SPINDLE	198	-0.19739081	-0.6422138	0.87279844	1	0.999
NOTCH_SIGNALING	31	-0.2139055	-0.6397593	0.9166667	1	0.999
E2F_TARGETS	190	-0.2648517	-0.622124	0.78019804	1	0.999
INFLAMMATORY_RESPONSE	198	-0.25689715	-0.6020838	0.9265873	0.99692255	0.999
IL6_JAK_STAT3_SIGNALING	87	-0.23669887	-0.56183934	0.9643564	0.99789304	1
PROTEIN_SECRETION	95	-0.16205812	-0.5151241	0.97137016	0.9930653	1
G2M_CHECKPOINT	185	-0.19927993	-0.5018363	0.89065605	0.9723166	1

### Immune Cell Infiltration

**Figure 6:**
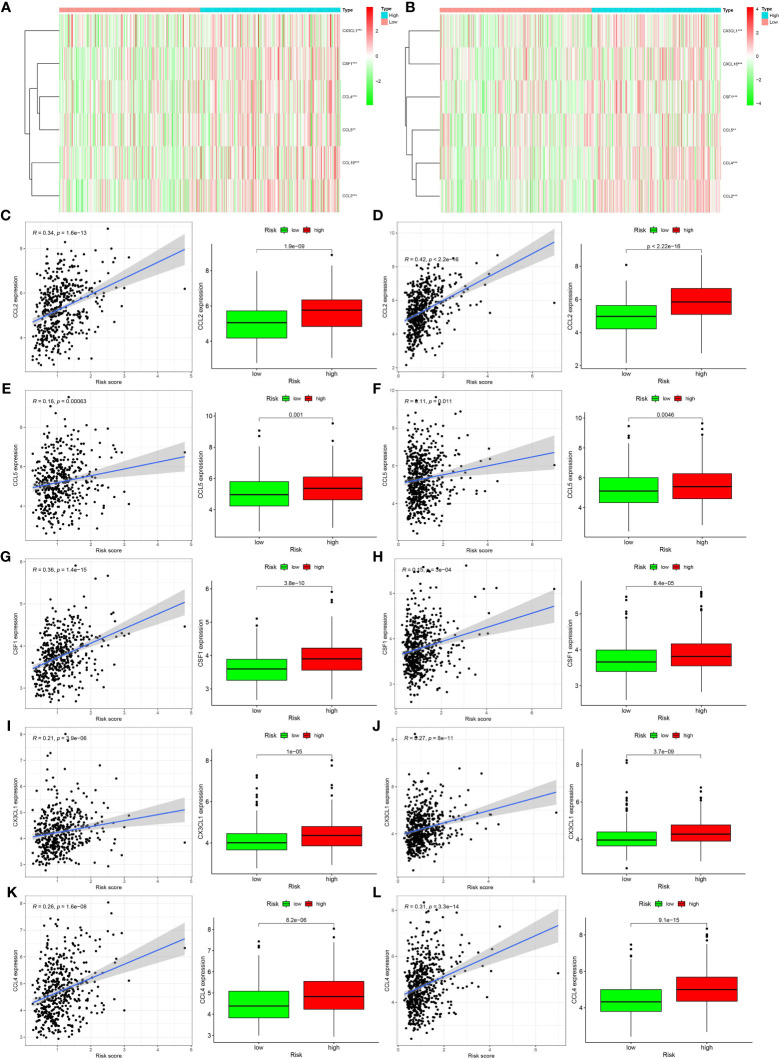
Relationships between genes regulating immune cell behavior and hypoxia risk. **(A, B)** Heat maps showing the expression levels of genes regulating activated CD4 memory T cells and M0 macrophages in different hypoxia risk groups (**p < 0.01; ***p < 0.001). **(C–L)** Scatter plots showing the correlations of the expression of four genes from The Cancer Genome Atlas and Gene Expression Omnibus databases with immune cell regulation, showing differences in the expression levels between the different hypoxia risk groups (p < 0.05). The blue line in each plot is a fitted linear model indicating the relationship between gene expression and the risk of hypoxia. Pearson coefficients were used to assess the correlation between the two factors. The box plots show the differences in the levels of gene expression between groups at risk of hypoxia (p < 0.05).

The results of the pathway enrichment analysis of hypoxia-related genes for both risk groups in TCGA and GEO databases showed the enrichment of pathways related to inflammation, immunity, and other factors. Based on these findings, we assessed the rate of immune cell infiltration in each risk group according to the constructed hypoxia-related gene model. **Figures 5C, D** shows the infiltration rate of immune cells in the high- and low-risk groups in TCGA and GEO databases, respectively. TCGA database showed differences in the infiltration rate of two types of immune cells in the high- and low-risk groups (**Figure 5E**; p < 0.05). In contrast, the rate of infiltration of 10 types of immune cells differed between the high- and low-risk groups in the GEO database (**Figure 5F**; p < 0.05). Among them, the infiltration rate of activated M0 macrophages differed between the high- and low-risk groups in both databases. Using the Tracking Tumor Immunophenotype online platform (http://biocc.hrbmu.edu.cn/TIP/index.jsp), we screened the immune-related genes for those that played important roles in the regulation of this immune cell type. Heat maps were then drawn to visualize the expression of these genes relative to that of hypoxia-related genes in the high- and low-risk groups of patients in TCGA and GEO databases (**Figures 6A, B**). The expression levels of *CCL2/4/5, CSF1*, and *CX3CL1* were significantly different between the high- and low-risk groups in both databases (p < 0.05). Next, we plotted the correlation curves between the expression levels of these four genes and the risk scores, which showed that the expression levels of these four genes were positively correlated with the patients’ risk score. We also observed differences in the expression levels of these four genes between the high- and low-risk groups (**Figures 6C–L**).

## Discussion

Hypoxia is a feature of tumor physiology specifically that of mechanisms associated with the acquisition of some malignant attributes, such as metastasis, invasion (7–9), and drug resistance (10). In these processes, hypoxia-related genes act on the corresponding pathways or on the regulating immune cells. Hypoxia-inducible factor (HIF) is activated during hypoxia. Some immunosuppressive factors, such as vascular endothelial growth factor, are HIF target genes, which affect both angiogenesis and immunosuppression (11). Hypoxia also results in upregulated *EGFR* expression, which promotes ligand-independent epidermal growth factor receptor signaling (12, 13). This process increases the rate of tumor glycolysis, resulting in metabolic competition (14). In our study, we screened for hypoxia-related genes in the gut and found that the core genes (*ALDOB*, *GPC1*, *ALDOC*, and *SLC2A3*) were closely related to patient prognosis. The rate of aldolase-B and fructose-bisphosphate B-driven fructose metabolism is significantly increased in patients with colon cancer and liver metastasis, and in those with colorectal villous polyps (15, 16). *GPC1* has also been shown to be overexpressed in various malignancies (17, 18). A recent study has reported *GPC1* enrichment in tumor-derived exosomes (17). In melanoma*, NME1* has been shown to inhibit metastasis by activating *ALDOC* transcription (19). Solute carrier family 2, member 3 can increase glucose uptake in anoxic cells and, thus, increase the rate of glycolysis. These findings suggest that hypoxia-related gene expression levels are closely related to tumor development and metabolism (20); therefore, they were included in our hypoxia-related gene model.

With a deepening understanding of the mechanisms of hypoxia, its influence on tumor prognosis is also increasingly being understood (21, 22). To further investigate the relationship between the expression of hypoxia-related genes and patient prognosis, we evaluated patient prognosis by taking the product sum of the expression levels and the coefficients of *ALDOB*, *GPC1*, *ALDOC*, and *SLC2A3* in TCGA and GEO databases as the risk score. The prognosis of patients in the high- and low-risk groups differed significantly; the survival rate of patients in the low-risk group was significantly higher than that of their counterparts. However, the ROC curve analysis, which aimed to evaluate the accuracy of the survival estimates, showed that the curves obtained from the GEO database corresponded poorly to the observed prognosis. Thus, we also analyzed the influence of other factors, including age, sex, TNM staging, and our proposed risk score, on patient prognosis. We concluded that our proposed risk score showed a good correspondence with patient prognosis. However, the results of the multivariate analysis in the GEO database were not statistically significant. This finding suggests that the risk score alone cannot be used as an independent prognostic factor. The samples in the GEO database were all colorectal adenocarcinoma samples that had been obtained in France. Because the tumor type and region are very specific, these samples may not accurately reflect the relationship between hypoxia and colon cancer prognosis. Moreover, the prognosis of colon cancer is related to disease stage and clinical, histological, genetic, and molecular factors, among others. These factors should be considered in future studies.

We next applied GSEA to identify pathways enriched in the high- and low-risk groups in the two databases. We found that most of the enriched pathways were related to inflammation, immune response, and apoptosis. Hypoxia and cell death in tumor tissue produce large amounts of cell debris and trigger the release of inflammatory factors, which can attract macrophages and monocytes, and can induce macrophage polarization. After polarization, macrophages secrete inflammatory factors (23). These findings suggest a close relationship between hypoxia, inflammation, and the immune response. In addition, some apoptosis-related genes, such as p53, which is a tumor suppressor gene, are closely related to tumor apoptosis. Nilay et al. (24) found that mutations in p53 in gastric and esophageal cancer cells can induce hypoxia signaling. This finding was confirmed in a study involving nude mice.

The results of many previous studies and those of the present study have shown that hypoxia can recruit immune cells into the tumor microenvironment. Hypoxia-induced tumor-derived cytokines, such as IL-10 and transforming growth factor-beta, can induce tumor-associated macrophages to differentiate into M2 macrophages with immunosuppressive effects (25). When monocytes are stimulated by inflammatory factors, such as interferon-gamma and lipopolysaccharide, they activate M1 macrophages, which can secrete inflammatory factors, such as IL-6 and tumor necrosis factor-alpha, and can phagocytize invasive pathogens and tumor cells (23, 26). Hypoxia can also lead to immune escape through the role of immune cells (5); for example, hypoxia can reduce T-cell activity (27). Hypoxia is closely related to immune cell function. In our study, we observed significant differences in the level of activated M0 macrophages between the high- and low-risk groups in TCGA database. In the GEO database, the level of infiltration of activated CD4 memory T cells and M0 macrophages, activated and resting mast cells, and neutrophils differed significantly between the high- and low-risk groups. Both databases showed significant differences in the infiltration rate of activated M0 macrophages between the high- and low-risk groups. When we screened the genes that regulated this immune cell type, we found different levels of *CCL2/4/5* and *CSF1*expression between the high- and low-risk groups. Further analyses confirmed the significant correlation between the expression level of each of these genes and the risk of hypoxia.

Three of these four genes encode chemokines, which play a chemotactic role in immune cells, such as natural killer cells and monocytes, which are closely related to tumor development. *CCL2* and *CCL5* play important roles in prostate cancer metastasis and drug resistance (28, 29), while *CCL4* is associated with the clinical characteristics of breast cancer (30). *CSF1* is expressed in almost all tumors (31, 32). It recruits macrophages other than alveolar macrophages through *CSF1* receptors and regulates their differentiation (31, 33). Macrophages, a major component of the tumor microenvironment, contribute to tumorigenesis by promoting angiogenesis, immunosuppression, invasion, and metastasis (34, 35).

In conclusion, hypoxia plays an important role in the tumor microenvironment. Screening for genes that can affect the rate of immune cell infiltration revealed a correlation between these genes and the hypoxia risk score. Our findings show that hypoxia-related genes can affect the prognosis of intestinal cancer and may play a role in immune infiltration in intestinal cancer. Analysis of the relationship between hypoxia and immune cells may improve immunotherapy and tumor treatment.

## Data Availability Statement

The original contributions presented in the study are included in the article/supplementary material. Further inquiries can be directed to the corresponding authors.

## Ethics Statement

All participants provided a signed informed consent.

## Author Contributions

The data analysis and original writing of the draft were conducted by LZ and SW. HX and NZ came up with the design and critical revision of the manuscript. The original writing of the draft and its editing were by YW, WZ, and YZ. All authors contributed to the article and approved the submitted version.

## Funding

Finance Department of Jilin (2018SCZWSZX-039); Finance Department of Jilin (JLSWSRCZX2020-083); Science and Technology of Jilin (20170623092TC-119).

## Conflict of Interest

The authors declare that the research was conducted in the absence of any commercial or financial relationships that could be construed as a potential conflict of interest.
